# Omalizumab versus cyclosporin-A for the treatment of chronic spontaneous urticaria: can we define better-responding endotypes?^☆^

**DOI:** 10.1016/j.abd.2022.03.003

**Published:** 2022-07-16

**Authors:** Emek Kocatürk, Emel Bülbül Başkan, Özlem Su Küçük, Mustafa Özdemir, Sinem Örnek, Pelin Kuteyla Can, Eda Haşal, Burhan Engin, Nilgün Atakan, Erkan Alpsoy

**Affiliations:** a Department of Dermatology, Koc University, School of Medicine, Istanbul, Turkey; b Department of Dermatology, Uludag University, School of Medicine, Bursa, Turkey; c Department of Dermatology, Bezmialem Vakif University, School of Medicine, Istanbul, Turkey; d Department of Dermatology, Istanbul Medipol University, School of Medicine, Istanbul, Turkey; e Department of Dermatology, Ankara Diskapi Yildirim Beyazit Training and Research Hospital, Ankara, Turkey; f Department of Dermatology, VM Medical Park Maltepe Hospital, Istanbul, Turkey; g Department of Dermatology, Istanbul University, Cerrahpasa School of Medicine, Istanbul, Turkey; h Department of Dermatology, Hacettepe University, School of Medicine, Ankara, Turkey; i Department of Dermatology, Akdeniz University, School of Medicine, Antalya, Turkey

**Keywords:** Biomarker, Chronic urticaria, Cyclosporine, Omalizumab

## Abstract

**Background:**

Chronic Spontaneous Urticaria (CSU) is characterized by recurrent wheals and/or angioedema for longer than 6-weeks. Guidelines recommend Omalizumab (Oma) as first-line and Cyclosporine-A (Cs-A) as second-line treatment in antihistamine resistant CSU. This step-wise algorithm might be time-consuming and costly.

**Objective:**

To determine indicators of response to Oma or Cs-A in CSU patients.

**Methods:**

We retrospectively analyzed data from seven centers in Turkey; the inclusion criteria for patients were to receive both Oma and Cs-A treatment (not concurrently) at some point in time during their follow-up. Clinical and laboratory features were compared between groups.

**Results:**

Among 110 CSU patients; 47 (42.7%) were Oma-responders, 15 (13.6%) were Cs-A-responders, and 24 (21.8%) were both Oma and Cs-A responders and 24 (21.8%) were non-responders to either drug. High CRP levels were more frequent in Cs-A-responders (72.7% vs. 40.3%; p = 0.055). Oma-responders had higher baseline UCT (Urticaria Control Test) scores (6 vs. 4.5; p = 0.045). Responders to both drugs had less angioedema and higher baseline UCT scores compared to other groups (33.3% vs. 62.8%; p = 0.01 and 8 vs. 5; p = 0.017). Non-responders to both drugs had an increased frequency in the female gender and lower baseline UCT scores compared to other groups (87.5% vs. 61.6%; p = 0.017 and 5 vs. 7; p = 0.06).

**Study Limitations:**

Retrospective nature, limited number of patients, no control group, the lack of the basophil activation (BAT) or BHRA (basophil histamine release assay) tests.

**Conclusions:**

Baseline disease activity assessment, which considers the presence of angioedema and disease activity scores, gender, and CRP levels might be helpful to predict treatment outcomes in CSU patients and to choose the right treatment for each patient. Categorizing patients into particular endotypes could provide treatment optimization and increase treatment success.

## Introduction

Chronic Spontaneous Urticaria (CSU) is defined as having wheals and or angioedema for longer than 6 weeks. The main effector cell in the pathophysiology of the disease is the mast cell, which degranulates after stimulation mainly via the high-affinity FcεR1 and leads to the release of a variety of mediators and results in the recruitment of cells such as basophils, eosinophils and T-lymphocytes.^1^, ^2^ It is widely accepted that autoimmune mechanisms play a major role in the activation of mast cells. In CSU, two types of autoimmunity are postulated; these are: 1) Type I autoimmunity (also called autoallergy) in which IgE autoantibodies and 2) Type IIb autoimmunity in which IgG autoantibodies against autoantigens activate mast cell receptors.^3^ The International Urticaria Guidelines recommend Omalizumab (Oma) as the first-line treatment in antihistamine resistant (patients who do not respond to four-fold doses of H1-antihistamines) CSU and Cyclosporine-A (Cs-A) is recommended in patients who fail on Omalizumab (Oma) treatment. Although this step-wise algorithm has been adopted, it might be time-consuming, costly, and might not be suitable for all CSU patients.^1^ There have been attempts to find biomarkers or clinical features which define response to treatment in CSU patients and; autologous serum test, basophil histamine release assay (BHRA), total IgE levels, d-dimer and CRP are some of the biomarkers that have been found to be associated with response to Oma or Cs-A treatment.^4^ However, many of the studies that have been performed to determine biomarkers focused on a certain drug which in this case is Oma or Cs-A, but there is a lack of studies that compare Oma-responders with Cs-A-responders. In this study, we aimed to determine if specific patient characteristics or laboratory markers could be used as indicators of Oma or Cs-A response in CSU patients.

## Methods

We retrospectively analyzed patient files from 7 centers in Turkey (Okmeydani Training and Research Hospital, Istanbul; Uludag University, School of Medicine, Bursa; Bezmialem Vakif University, School of Medicine, Istanbul; Istanbul Medipol University, School of Medicine, Istanbul; Istanbul University, Cerrahpasa School of Medicine, Istanbul; Hacettepe University, School of Medicine, Ankara; Akdeniz University, School of Medicine, Antalya) that are experienced in treating CSU patients. The diagnosis of CSU was made based on physical examination and patient history. Patients were considered antihistamine resistant when they did not respond to four-fold doses of H1-antihistamines. The inclusion criteria for CSU patients were to:

1 Have chronic spontaneous urticaria for longer than 6-weeks,
2 To be aged over 18-years,
3 Not to be pregnant and breastfeeding,
4 To receive a trial of both Oma and Cs-A treatment (not concurrently) during their disease course for at least 3-months to decide if there is a response to treatment or not,
5 To include Urticaria Control Test (UCT) scores and sufficient clinical and laboratory data in the files.

Exclusion criteria were to:

1 Chronic inducible urticaria,
2 Patients under 18 years of age,
3 Take both Cs-A and Oma at the same time.

Response to treatment was defined by UCT scores (UCT ≥ 12; under control). Four groups of patients were defined according to treatment responses:

1 Patients who respond to only Oma (do not respond to CsA) (Oma-resp^+^),
2 Patients who respond to only Cs-A (do not respond to Oma) (Cs-A-resp^+^),
3 Patients who respond to both Oma and Cs-A (Oma-Cs-A-resp^+^),
4 Patients who do not respond to either drug (Oma-Cs-A-resp^-^).

We performed a comparison among all response groups in terms of features such as age, gender, family history, presence of angioedema, disease duration, accompanying inducible Urticaria (CIndU), baseline UCT scores and laboratory markers such as Autologous Serum Skin Test (ASST), high CRP, high ESR, eosinopenia, basopenia, low total IgE levels, presence of autoimmune thyroid disease as well as anti-Thyroid Peroxidase (anti-TPO) and anti-Thyroglobuline (anti-TG) positivity.

Ethics approval was taken from the coordinating center Okmeydani Training and Research Hospital’s Ethics Committee (date 23.05.2017; issue 98-667).

### Definitions

High levels of CRP: ≥5 mg/L,

High levels of ESR: ≥20 mm/h,

Basopenia <0.01 × 10^9^/L,

Eosinopenia <0.05 × 10^9^/L,

Low total IgE levels <43 IU/mL,

Autoimmune Thyroid disease (AIT): established diagnosis of AIT found in patient charts (not from only anti-Thyroid Peroxidase (TPO) /anti-Thyroglobuline (TG) positivity),

High disease activity UCT < 6.

### Statistical analysis

We described baseline characteristics with means and Standard Deviations (SD) for continuous variables and frequencies and percentages for categorical variables. Pearson’s Chi-Squared and Fisher's exact test were used for group comparison of categorical variables. Mann-Whitney *U* test was used for the comparisons of continuous variables between groups. Only low IgE could be claimed to have an association with Cs-A treatment outcomes^5^ that would confound the associations presented in previous papers though it is not fully confirmed. We, therefore, used multinominal logistic regression models to calculate low IgE (lower than 43)-adjusted ORs (aAORs) to examine associations between Cs-A treatment outcomes for CSU; p-values lower than 0.05, two-sided, were considered statistically significant.

## Results

**Figure 1:**
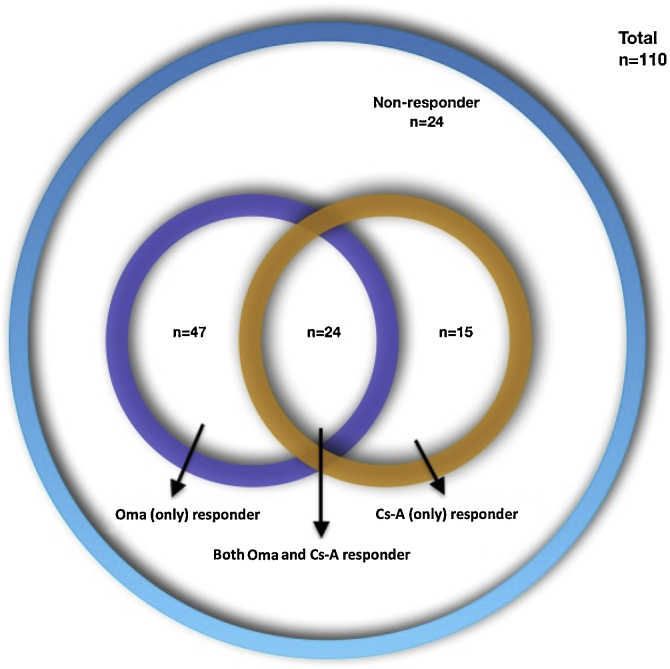
The distribution of four types of treatment responses.

One-hundred-ten CSU patient files were included from 7 centers (74 female [67.3%], mean age 40.98 ± 12.37; range: 16-81 years, mean disease duration 57.21 ± 69.31; range: 6‒402 months). The demographic characteristics of the patients are presented in Table 1. Four types of treatment responses were distributed as follows: 47 (42.7%) Oma-resp^+^, 15 (13.6%) Cs-A-resp+, 24 (21.8%) Oma-Cs-A-resp + and 24 (21.8%) Oma-Cs-A-resp- (Fig. 1). The laboratory findings of the patients are presented in Table 2. Statistically significant differences, as well as statistically not significant but more frequent features, are presented in Table 3.

**Table 1:** Demographic and clinical characteristics of the patients.

Demographic and clinical parameters	Total (n = 110, %)	Oma responders (n = 47, 42.7%)	Cs-A responders (n = 15, 13.6%)	Both Oma and Cs-A responders (n = 24, 21.8%)	Non-responders (n = 24, 21.8%)
Age in years					
Min‒Max (Median)	16‒81 (39)	27‒81 (42)	16‒73 (37)	21‒66 (34.5)	20‒69 (37)
Mean ± SD	40.98 ± 12.37	43.32 ± 10.67	40.20 ± 16.52	37.79 ± 12.18	40.08 ± 12.64
Gender n (%)					
Male	36 (32.7%)	18 (38.3%)	6 (40%)	9 (37.5)	3 (12.5)
Female	74 (67.3%)	29 (61.7%)	9 (60%)	15 (62.5%)	21 (87.5%)
Accompanying CINDU n (%)	n = 99	n = 43	n = 15	n = 17	n = 24
	21 (21.2%)	9 (20.9%)	3 (20%)	3 (17.6%)	6 (25%)
Disease duration (months)					
Min‒Max (Median)	0‒402 (30)	2‒300 (30)	3‒120 (36)	0‒264 (25)	5‒402 (36)
Mean ± SD	57.21 ± 69.31	60.32 ± 71.26	45.53 ± 38.33	51.46 ± 63.72	64.17 ± 86.37
Angioedema, n (%)	62 (56.4%)	27 (57.4%)	10 (66.7%)	8 (33.3%)	17 (70.8%)
Baseline UCT	n = 83	n = 41	n = 8	n = 16	n = 18
Min‒Max (Median)	0‒16 (6)	1‒16 (6)	2‒9 (3)	3‒16 (8)	0‒11 (5)
Mean ± SD	7 ± 4.34	7.44 ± 4.65	4.75 ± 3.06	9.12 ± 3.81	5.11 ± 3.53
UCT ≥ 6	46 (55.4%)	23 (56.1%)	3 (37.5%)	13 (81.2%)	7 (38.9%)
UCT < 6	37 (44.6%)	18 (43.9%)	5 (62.5%)	3 (18.8%)	11 (61.1%)
Positive family history n (%)	11 (10%)	2 (4.3%)	3 (20%)	3 (12.5%)	3 (12.5%)

CINDU, Chronic Inducible Urticaria; UCT, Urticaria Control Test.

**Table 2:** Laboratory findings of the patients shown for each group.

Laboratory finding	Total (n = 110, %)	Oma (only) responder (n = 47, 42.7%)	Cs-A (only) responder (n = 15, 13.6%)	Both oma and Cs-A responder (n = 24, 21.8%)	Non-responder (n = 24, 21.8%)
High levels of CRP	n = 88	n = 38	n = 11	n = 21	n = 18
	39 (44.3%)	16 (42.1%)	8 (72.7%)	7 (33.3%)	8 (44.4%)
High levels of ESR	n = 99	n = 44	n = 13	n = 21	n = 21
	39 (39.4%)	15 (34.1%)	8 (61.5%)	9 (42.9%)	7 (33.3%)
Eosinopenia	n = 54	n = 23	n = 10	n = 9	n = 12
	3 (5.6%)	0	2 (20%)	0	1 (8.3%)
Basipodia	n = 102	n = 46	n = 12	n = 24	n = 20
	4 (3.9%)	1 (2.2%)	2 (16.7%)	0	1 (5%)
ASST positivity	n = 77	n = 36	n = 7	n = 17	n = 17
	44 (57.1%)	19 (52.8%)	5 (71.4%)	10 (58.8%)	10 (58.8%)
Serum total IgE levels (IU/mL)	2,03‒6350 (167)	3,28‒6350 (169)	2,03‒1282 (280)	6,4 3‒16 06 (150)	9‒1434 (172)
	370.65 ± 727.22	476.35 ± 1000.81	343.27 ± 392.27	252.58 ± 368.81	271.05 ± 328.70
Serum total IgE levels lower than 43 (IU/mL)	n = 94	n = 43	n = 12	n = 18	n = 21
	23 (24.5%)	9 (20.9%)	5 (41.7%)	3 (16.7%)	6 (28.6%)
Autoimmune thyroid disease	n = 81	n = 35	n = 9	n = 20	n = 17
	17 (21%)	8 (22.9%)	2 (22.2%)	2 (10%)	5 (29.4%)
High levels of anti-TPO antibody n (%)	n = 72	n = 29	n = 8	n = 19	n = 16
	25 (34.7%)	11 (37.9%)	4 (50%)	4 (21.1%)	6 (37.5%)
High levels of anti-TG antibody n (%)	n = 66	n = 25	n = 8	n = 19	n = 14
	18 (27.3%)	8 (32%)	2 (25%)	3 (15.8%)	5 (35.7%)
Positive H.pylori antigen in stool n (%)	n = 87	n = 41	n = 12	n = 19	n = 15
	31 (35,6%)	13 (31,7%)	4 (33,3%)	7 (36,8%)	7 (46,7%)

anti-TG, anti-Thyroglobulin antibody; anti-TPO, anti-Thyroid Peroxidase antibody; CRP, C-Reactive Protein; ESR, Erythrocyte Sedimentation Rate; *H. pylori*, *Helicobacter pylori*; ASST, Autologous Serum Skin Test.

**Table 3:** Demonstration of statistically significant and statistically no-significant but more frequent features observed between the treatment response groups.

Treatment group	p < 0.05	p > 0.05
Oma (only) responders vs. Cs-A (only) responders	None	More frequent in Cs-A responders: Positive ASST, positive family history, high CRP, basopenia, eosinopenia, high ESR, low total IgE levels, anti-TPO positivity, lower baseline UCT scores
Oma responders vs. oma-non-responders	Higher in oma responders: Baseline UCT score	More frequent in Oma-responders: Male gender, lower rates of ASST positivity, lower rates of family history, lower frequency of high CRP, lower frequency of eosinopenia, lower frequency of basopenia, lower frequency of high ESR levels, lower frequency of angioedema, lower frequency of autoimmune thyroid disease, higher frequency of total IgE levels greater than 43
Cs-A responders vs. Cs-A-non-responders	More frequent in Cs-A responders: High CRP	More frequent in Cs-A responders: Positive family history, positive ASST, eosinopenia, basopenia, high ESR levels, lower total IgE levels, anti-TPO positivity, lower baseline UCT scores
Responders to both drugs vs. other three groups	More frequent in both drug responders: Lower angioedema frequency, higher baseline UCT	Less frequent in both drug responders: High CRP, eosinopenia, basopenia, autoimmune thyroid disease, anti-TPO positivity, anti-TG positivity
Non-responders to either drug vs. other three groups	Higher frequency in non-responders: Female gender, lower baseline UCT	Higher frequency in non-responders: Angioedema, autoimmune thyroid disease, anti-TG positivity, *H.pylori*
Cs-A responders vs. Cs-A-non-responders	More frequent in Cs-A responders: High CRP	More frequent in Cs-A responders: Positive family history, positive ASST, eosinopenia, basopenia, high ESR levels, lower total IgE levels, anti-TPO positivity, lower baseline UCT scores

anti-TPO, anti-Thyroid Peroxidase antibody; anti-TG, anti-Thyroglobulin antibody; ASST, Autologous Serum Skin Test; CRP, C-Reactive Protein; Cs-A, Cyclosporine-A; ESR, Erythrocyte Sedimentation Rate; *H.pylori*, *Helicobacter pylori*; Oma, Omalizumab; UCT, Urticaria Control Test.

### No significant differences between Oma-responders and Cs-A-responders but some features are more prevalent in Cs-A-responders

When a comparison was performed between patients who responded only to Oma (n = 47) and only to Cs-A (n = 15), there were no statistically significant differences between the groups in terms of the clinical and laboratory parameters. But positive ASST (52.8% vs. 71.4%; p = 0.47), positive family history (4.3% vs. 20%; p = 0.086), high levels of CRP (42.1% vs. 72.7%; p = 0.074), basopenia (2.2% vs. 16.7%; p = 0.10), eosinopenia (0 vs. 20%; p = 0.08), high levels of ESR (34.1% vs. 61.5%; p = 0.07), low total IgE levels (20.9% vs. 41.7%; p = 0.259), anti-TPO positivity (37.9% vs. 50%; p = 0.69) and lower baseline UCT scores (6 vs. 3; p = 0.122) were more frequent in Cs-A-resp^+^ even though these were not statistically significant.

### Oma-responders have higher baseline UCT scores compared to Oma-non-responders

When Oma-resp^+^ (n = 47) were compared to Oma-resp^-^ (Oma-Cs-A-resp^-^ and Cs-A-resp^+^; n = 39), the only statistically significant parameter was baseline UCT score which was 6 vs. 4.5 in Oma-resp^+^ vs. Cs-A-resp^+^ and Oma-Cs-A-resp^-^, respectively (p = 0.045). Other features which were observed without statistical significance in Oma-resp^+^ were higher rate male gender (38.3% vs. 23.1%; p = 0.13), lower ratio of ASST positivity (52.8% vs. 62.5%; p = 0.45), lower ratio of family history (4.3% vs. 15.4%; p = 0.13), lower frequency of high CRP (42.1% vs. 55.2%; p = 0.29), lower frequency of eosinopenia (0% vs. 13.6%; p = 0.10), lower frequency of basopenia (2.2% vs. 9.4%; p = 0.30), lower frequency of high ESR levels (34.1% vs. 44.1%; p = 0.36), lower frequency of angioedema (57.4% vs. 69.2%; p = 0.26), lower frequency of autoimmune thyroid disease (22.9% vs. 26.9%; p = 0.71) and higher frequency of total IgE levels greater than 43 (79.1% vs. 66.7%; p = 0.22).

### High CRP levels are more frequent in Cs-A-responders compared to Cs-A-non-responders

The only statistically significant difference between Cs-A-resp^+^ (n = 15) and Cs-A-resp- (Oma-Cs-A-resp^-^, Oma-resp^+^ and Oma-Cs-A-resp^+^; n = 95) was high levels of CRP which was more frequent among Cs-A-resp^+^ (72.7% vs. 40.3%; OR = 3.96, 95% CI 0.97‒16.1; p = 0.05) and having high CRP levels increased the chance of favorable treatment response to Cs-A by 6.1 after adjusting for low levels of total IgE (aOR = 6.1; 95% CI 1.17‒31.8; p = 0.032).

Positive family history (20% vs. 8.4%; p = 0.172), positive ASST (71.4% vs. 55.7%; p = 0.692), eosinopenia (20% vs. 2.3%; p = 0.085), basopenia (16.7% vs. 2.2%; p = 0.067), high ESR levels (61.5% vs. 36%; p = 0.08), lower total IgE levels (41.7% vs. 22%; p = 0.159), anti-TPO positivity (50% vs 32.8%; p = 0.436), lower baseline UCT scores (3 vs. 7; p = 0.107) were more frequent without statistical significance in Cs-A-resp^+^.

### Female gender and lower baseline UCT were more frequent in non-responders of either drug

Non-responders of either drug was compared to all responder groups (Oma-resp^+^, Cs-A-resp^+^ and Oma-Cs-A-resp^+^) and the comparison revealed that the majority of Oma-Cs-A-resp^-^ were females (21;87.5% vs. 53;61.6%; p = 0.017) and baseline UCT was lower in Oma-Cs-A-resp- (5 vs. 7; p = 0.06).

### Responders to both drugs had less angioedema and higher baseline UCT

Oma-Cs-A-resp^+^ were compared to the rest of the patients (Oma-resp^+^, Cs-A-resp^+^ and Oma-Cs-A-resp^-^). Angioedema frequency was lower (8;33.3% vs. 54;62.8%; p = 0.01) and baseline UCT was higher in Oma-Cs-A-resp^+^ (8 vs. 5; p = 0.017).

## Discussion

In this study, we found that in a subpopulation of antihistamine refractory CSU patients who received both Oma and Cs-A any time during their follow up, the majority (forty percent) responded to Oma, the minority (ten percent) responded to Cs-A, while twenty percent responded to both Oma and Cs-A and another twenty percent did not respond to either drug (Summary of findings in Table 4). These findings are interesting owing to the reports of high response rates of CSU patients to Cs-A treatment which ranged between 38% to 100%.^6^ We would have expected more patients to be responding to Cs-A treatment however after careful examination of clinical and laboratory findings of the studied patient population, t 65% of our patient population had high total IgE levels (higher than 100 IU/mL) which have been reported to be a feature of type I autoimmune CSU and an unfavorable factor for Cs-A response.^3^, ^5^ Maurer et al.^3^ described some features of type I versus type IIb autoimmune CSU and pointed out that the presence of auto-IgE (eg; against TPO, IL-24, ds-DNA), higher rates of concomitant allergic diseases, normal or high total IgE levels and high response rate to oma favors type I autoimmune CSU while the presence of auto-IgG (against IgE, FcεRI), higher disease activity, longer disease duration, higher rates of concomitant autoimmune diseases, lower total IgE levels, higher rates of eosinopenia and basopenia, higher levels of CRP, higher rates of ANA positivity, low response rate or slower response to Oma and good response to immunosuppressive treatment. Based on this endotype classification, we can explain why the response rate to Cs-A is low in the studied patient population; we believe the low frequency of patients with eosinopenia, basopenia, low IgE levels, high CRP levels, and high disease severity; but high frequency of patients with high total IgE levels show that the majority of the patient population are composed of type I autoimmune CSU and therefore more prone to respond to Oma treatment.

**Table 4:** What is already known about the topic, what does this article add?

What is already known about the topic?	What does this article add?
Autoimmunity in CSU has been classified into type 1 autoimmune (IgE type-autoallergic) and type 2b autoimmune (IgG type) mechanisms. These two types have been associated with different clinical and laboratory features and treatment response patterns. Type 1 autoimmune urticaria is associated with IgE antibodies against autoantigens, higher rates of concomitant allergic diseases, normal or high total IgE levels and high response rates to omalizumab. Type 2b autoimmune urticaria is associated with IgG antibodies against autoantigens, BHRA, BAT, ASST positivity, more severe disease with longer duration, higher rates of concomitant autoimmune diseases, higher rates of basopenia and eosinopenia, lower total IgE levels, higher CRP levels, higher ANA positivity, low response rates to antihistamines and omalizumab, higher response rates to immunosuppressive therapy.	We found two distinct patterns of CSU endotypes according to their treatment response patterns which are compatible with the two different autoimmune urticaria endotypes; Oma responders tended to be more male gender with a lower disease activity (higher baseline UCT scores) and with lower rates of ASST positivity, lower rates of family history, lower CRP levels, lower rates of basopenia and eosinopenia, lower ESR levels, lower frequency of angioedema, lower frequency of autoimmune thyroid disease and higher total IgE levels. These are features that have been linked to type 1 (IgE type) autoimmunity. Cs-A responders had higher CRP levels and higher frequency of positive family history, positive ASST, eosinopenia, basopenia, higher ESR levels, higher frequency of anti-TPO positivity, lower total IgE levels and higher disease activity (lower baseline UCT scores). These are features that have been linked to type 2b (IgG type) autoimmunity.
Biomarkers for Oma response has been reported to be high levels of total IgE, ASST negativity, BHRA negativity, lack of basophil CD203c-upregulating activity, high expression of basophil FcεRI and lower IL-31 levels while biomarkers for cyclosporine response has been reported to be BHRA positivity, higher CRP levels, low total IgE levels, ASST positivity, low D-dimer levels, high disease activity and short disease duration	Non-responders to either drugs are more female with higher disease activity and with more angioedema, autoimmune thyroid disease, thyroid autoantibodies and *H.pylori* positivity.

anti-TPO, anti-Thyroid Peroxidase antibody; anti-TG, anti-Thyroglobulin antibody; ASST, Autologous Serum Skin Test; BAT, Basophil Activation Test; BHRA, Basophil Histamine Release Assay; CRP, C-Reactive Protein; Cs-A, Cyclosporine-A; CSU, Chronic Spontaneous Urticaria; ESR, Erythrocyte Sedimentation Rate; *H.pylori, Helicobacterpylori*; Oma, Omalizumab; UCT, Urticaria Control Test.

Searching for biomarkers that predict response to a particular treatment has been a matter of interest and many publications reported some biomarkers might be helpful in determining response to certain treatments in CSU. For Oma, high levels of IgE, ASST negativity, BHRA negativity, the lack of basophil CD203c-upregulating activity, high expression of basophil FcεRI and lower IL-31 levels have been reported to be biomarkers for good treatment response, while for cyclosporine, BHRA positivity, higher CRP levels, low IgE levels, ASST positivity, low D-dimer levels, high disease activity, and short disease duration were indicative of good treatment response.^4^, ^7^ In the present study population, the authors could not find a statistically significant difference between Oma and Cs-A responders with respect to the comparison parameters but this might be the result of the imbalanced distribution of patients with certain parameters; as mentioned before the authors have a lower percentage of patients with good response to Cs-A treatment that could have resulted in a limitation of statistical analysis; that is mainly due to the choice of omalizumab as a first-line treatment in antihistamine refractory patients. Comparison between groups showed positive ASST, positive family history, high levels of CRP, basopenia, eosinopenia, high levels of ESR, low total IgE levels, anti-TPO positivity, and lower baseline UCT scores were more frequent in Cs-A responders than Oma-responders. These findings again refer to the importance of endotypes of CSU; the mentioned parameters associated with cyclosporine response are also features of type IIb autoimmune CSU. Type IIb autoimmune urticaria is defined by IgG anti‐IgE or FcεRI, a positive Basophil Activation Test (BAT), and a positive ASST and has been shown to respond better to Cs-A treatment.^8^, ^9^, ^10^, ^11^ The authors also found that patients with high CRP levels responded better to Cs-A, which is a finding, reported in previous studies.^12^ Furthermore, the authors observed that low total IgE levels were more frequent in Cs-A responders as was reported by Santiago et al.^5^ Similar to the findings of Hollander et al.^11^ who reported that higher initial severity predicted a successful response to treatment with cyclosporine, Cs-A responders in the present study had lower UCT scores. Even though Cs-A responders in the present study had a higher frequency of ASST positivity, the evidence of ASST being a potential predictor of treatment response for Cs-A has not been conclusive.

Omalizumab responders in the present study population were found to have a significantly higher baseline UCT score than omalizumab non-responders, which pointed out to milder disease activity and was also reported by Salman et al.^13^ Presence of angioedema,^14^ female gender,^15^ high CRP levels,^16^ ASST positivity,^17^ eosinopenia and basopenia ^18^ has been reported to be associated with poor response to omalizumab. Higher serum total IgE levels are currently accepted as an established biomarker of omalizumab response^14^ even though the authors were not able to show this in a statistically significant manner in the present study.

One out of five patients in the present study showed a response to both drugs and these patients were found to have less angioedema and higher baseline UCT scores (lower baseline disease activity) which are features of lower disease activity. It is anticipated to have a favorable response to treatment in patients with lower disease activity, however, it would be illuminative if the authors could perform testing for both IgE autoantibodies and IgG autoantibodies and determine which type of autoimmunity these patients have. Then the authors would hypothesize that these patients might have both types of autoantibodies and therefore both types of autoimmunity. The idea of the presence of such patients comes from the observations of increasing reports of patients who respond to treatment with Oma and Cs-A together. But the patients in these reports do not seem to respond to treatment with either Oma or Cs-A alone but to treatment with a combined regimen; reflecting patients with high disease activity and possibly with two types of autoimmunity.^19^, ^20^, ^21^ Two types of autoimmunity existing together have been demonstrated in a recent clinical study by Asero et al.^22^ who showed the co-existence of IgE and IgG autoantibodies to high- and low-affinity IgE receptors (FcεRI and FcεRII), tissue factor and thyroglobulin, particularly in late responders to omalizumab.

Twenty percent of the patients in this study did not respond to treatment with either Oma or Cs-A. This refractory patient population included more females and more patients with lower baseline UCT scores. These features have also been linked to refractory disease in previous reports; the female gender has been reported to be a predictor of longer time to remission,^23^ anti-histamine resistance,^24^ and higher frequency of recurrence after omalizumab treatment.^25^ Lower baseline UCT scores have been associated with a lower response rate to omalizumab and a higher need for omalizumab up dosing.^26^ Despite not being statistically significant, the refractory patients in the present study had a higher rate of *H.pylori* positivity even though the information on *H.pylori*eradication rates in these patients is lacking. This emphasizes the importance of evaluating and treating comorbid conditions in CSU patients.

The only study that is in some ways similar to ours is the one published by Sánchez et al. who reported that out of 88 patients in their study; 26/88 (29.5%) responded to Cs-A, 41/88 (46.5%) responded to Oma, 16/88 (18.2%) responded when Cs-A and Oma were combined and only 5 (5.6%) patients were left without response to treatment.^19^ Response rates to omalizumab were similar to the present study and were also similar to the complete response rate reported by the clinical trials and real-life studies of omalizumab, which is around 40%‒50%.^27^ But the rate of non-responders of both drugs seems to be very much lower than in the present study which might be attributed to our preference for not including patients who used both omalizumab and cyclosporine at the same time. If these refractory patients were treated with both drugs, the rate of non-responders could have been decreased.

Why these patients do not respond to both omalizumab and cyclosporine needs to be explored in larger prospective trials, which assess the profile of patients with detailed laboratory examinations including IgG type and IgE type autoantibodies, BAT/BHR testing as well as other inflammatory mediators both in the skin and peripheral blood. These patients might have a different underlying pathomechanism including involvement of complement components, infiltrates in the skin composed of eosinophils, or T-lymphocytes producing other inflammatory cytokines such as IL-4, IL-5, IL-23, IL-17 or IL-31, or other mechanisms including angiogenesis, coagulation, and vascular dysregulation.^28^ There are many different ranges of therapeutic options for this refractory group of patients which are under clinical trials or in progress of development including dupilumab, fenebrutinib, benralizumab, and anti-siglec-8.^2^, ^29^

Limitations of the present study are its retrospective nature, which did not include the same number of patients in the outlined four groups, not including patients who were treated with both Cs-A and Oma, having a low number of patients, having no control group, and the lack of BAT or BHRA testing. The cross-sectional nature of the study led to a study population, which might not be reflective of the general urticaria population, but the multicenter nature of the study might have mitigated this effect.

## Conclusion

Categorizing patients into endotypes could be helpful in predicting treatment responses and could aid in choosing the right treatment option for the particular CSU patient. For the ultimate care of patients, determining disease activity at baseline and paying attention to the laboratory markers such as CRP, total IgE and IgG-anti-TPO as well as clinical markers such as female gender and angioedema might be of special importance.

## Funding sources

None.

## Authors' contributions

Emek Kocaturk: Substantial contributions to the study concept and design; Acquisition, analysis, and interpretation of data; Critical review of the literature; Involvement in drafting the manuscript; writing the manuscript; Final approval of the version of the manuscript.

Emel Bülbül Başkan: Data collection, or analysis and interpretation of data; Critical review of important intellectual content; Final approval of the final version of the manuscript.

Özlem Su Küçük: Data collection, or analysis and interpretation of data; Critical review of important intellectual content; Final approval of the final version of the manuscript.

Mustafa Özdemir: Data collection, or analysis and interpretation of data; Critical review of important intellectual content; Final approval of the final version of the manuscript.

Sinem Örnek: Data collection, or analysis and interpretation of data; Effective participation in the research guidance; Final approval of the final version of the manuscript.

Pelin Kuteyla Can: Data collection, or analysis and interpretation of data; Effective participation in the research guidance; Final approval of the final version of the manuscript.

Eda Haşal: Data collection, or analysis and interpretation of data; Effective participation in the research guidance; Final approval of the final version of the manuscript.

Burhan Engin: Data collection, or analysis and interpretation of data; Critical review of important intellectual content; Final approval of the final version of the manuscript.

Nilgün Atakan: Data collection, or analysis and interpretation of data; Critical review of important intellectual content; Final approval of the final version of the manuscript.

Erkan Alpsoy: Data collection, or analysis and interpretation of data; Critical review of important intellectual content; Final approval of the final version of the manuscript.

## Conflicts of interest

None declared.
